# Research of antimicrobial resistance and its associated genes distribution in *Escherichia coli* from diarrheic calves in the Ulagai region of China

**DOI:** 10.3389/fvets.2025.1685829

**Published:** 2025-11-24

**Authors:** Wei Gao, Xinying Zhang, Miao Sun, Dongxu Han, Jianing Wang, Yue Li, Sanren, Lidong Yu, Fang Gui, Lei Guo, Nimalaxi, Zi Wang, Kai Liu

**Affiliations:** 1 College of Animal Science and Technology, Inner Mongolia Minzu University, Tongliao, China; 2 Tongliao Agricultural and Animal Product Quality Safety Center, Tongliao, China; 3 Animal Disease Prevention and Control Center of Wulagai Administrative Region, Tongliao, China; 4 Inner Mongolia Engineering Technology Research Center for Prevention and Control of Beef Cattle Diseases, Tongliao, China; 5 Beef Cattle Industry School of Inner Mongolia Autonomous Region, Tongliao, China

**Keywords:** *Escherichia coli*, calf diarrhea, gut microbiota, resistance genes, whole-genome sequencing

## Abstract

As a conditional pathogenic bacterium, *Escherichia coli* is a major contributor to infect calf diarrhea. It has attracted extensive attention due to antimicrobial resistance (AMR) and pathogenicity. To elucidate the AMR profiles and resistance-related genes in *E. coli* isolated from calf diarrhea samples in the Ulagai region *E. coli* was isolated and identified from samples of calf feces using *E. coli* chromogenic medium, Gram staining, and 16S rRNA sequencing. The antimicrobial susceptibility was tested using the Kirby-Bauer disk diffusion method. Resistance genes were analyzed using PCR. Additionally, strains showing severe multidrug resistance were selected for whole-genome sequencing. Multidrug resistance was observed in all 50 isolated *E. coli* strains. They were resistant to bacitracin, and 82% were resistant to gentamicin. Strains 24, 27, 36, and 15 exhibited particularly high levels of resistance. Analysis of resistance-related genes detected over 90% resistance associated with *TEM-1* and *tetR* and over 80% for *CTXM-55*, *QacH*, *strB*, and *floR*, *sul2* was observed in 100% of the isolates. Four strains indicated genome sizes of 5,144,828 bp, 4,798,224 bp, 4,813,249 bp, and 5,450,201 bp, respectively, harboring 5, 3, 6, and 2 plasmids. Prediction of antibiotic resistance genes revealed that the isolates contained numerous resistance genes, strain 27 carried the highest number (148 in total). All strains isolated from diarrheic calves exhibited multidrug resistance and carried numerous resistance genes. Furthermore, the observation of abundant mobile genetic elements in the strains increases the risk of horizontal gene transfer of resistance genes, indicating the severity of issues faced by clinical prevention and control measures.

## 1 Introduction

Diarrhea in newborn calves is common, typically occurring within the first few weeks after birth. It is associated with high mortality rates and has significant effects on the economic efficiency and development of the farming industry throughout the world (1–4). Both infectious and non-infectious factors can contribute to calf diarrhea, including poor management practices, the quality of animal nutrition, and the health status of the dam. However, the major cause is pathogenic infection (5–8).

*E. coli* is a commensal gut bacterium and a conditional pathogen. Under normal conditions, it contributes to the maintenance of intestinal homeostasis in the host. However, situations of reduced host immunity or microbiota imbalances can activate the expression of virulence genes, transforming it into a pathogen that causes diarrhea or even systemic infection in calves (9–11). Animal husbandry has relied heavily on antimicrobials for disease prevention and control. However, the excessive and inappropriate use of antibiotics in livestock and poultry farming has led to the emergence and spread of bacterial resistance. This resistance is associated with increased abundance of antibiotic resistance genes (ARGs) in pathogens, which not only severely influences the effective prevention and treatment of disease but also poses a serious threat to global public health security (12–14). As a potential source, intermediate vector, and important reservoir of ARGs, *E. coli* plays a crucial role in the dissemination of bacterial resistance (15).

Horizontal gene transfer (HGT) is a key factor influencing the spread of ARGs among bacteria. Mobile genetic elements (MGEs), such as plasmids, transposons, and integrons, promote the dissemination of ARGs through transduction, transformation, and conjugation (16, 17). These elements carry various genes related to microbial functions. Plasmids, which form part of the bacterial genome, contain genetic information that can be self-transferred via conjugation or assisted by other genomic elements, and are the primary mediators of ARG transmission (18). Bacterial integrons are genetic determinants containing components of site-specific recombination systems that can recognize and capture mobile gene cassettes, thereby also playing a significant role in mediating antibiotic resistance (19). However, integrons lack the capability for autonomous transfer and rely instead on associations with transposons and/or conjugative plasmids, utilizing insertion sequences (IS) within these elements as vectors for intra- or interspecies transmission (20, 21). There are five classes of integrons involved in the transfer of ARGs, of which Class I and II integrons are most commonly found in clinical isolates (22). Recurrent antimicrobial resistance (AMR) in bacteria represents a significant challenge in many developing countries. MGEs not only mediate HGT of ARGs but also drive the diversification of AMR bacteria, exacerbating the problem of resistance through this dual role.

This study aims to investigate the AMR characteristics of *E. coli* isolated from diarrheic calves in the Ulagai region of China. The investigation involves the isolation and identification of resistant strains, assessment of antimicrobial susceptibility, and analysis of resistance-related genes, thereby providing a preliminary assessment of the AMR status of *E. coli* from diarrheic calves in this region. The findings will provide a basis and guidance for clinical treatment and disease control in diarrheic calves in Ulagai and establish a foundation for further research into the transmission of bacterial resistance.

## 2 Materials and methods

### 2.1 Sample collection and strain isolation

From March to July 2023, 121 fecal samples were collected aseptically from 1 to 2-week-old diarrheic Simmental calves in the Ulagai region of Inner Mongolia. All fecal samples were collected non-repetitively from calves exhibiting diarrhea symptoms for the first time and without prior antibiotic treatment. The fecal samples were inoculated onto *E. coli* chromogenic medium and incubated at 37 °C for 12–18 h. Distinctive colored single colonies were selected and repeatedly streaked for purification. The isolated strains were identified using Gram staining and 16S rRNA sequencing. Genomic DNA was extracted from the isolates using a bacterial genomic DNA extraction kit (TransGen Biotech, Beijing, China), followed by PCR amplification with 16S rRNA primers. The amplification products were sequenced by Sangon Biotech (Shanghai) Co., Ltd., and analyzed using the BLASTN program on the National Center for Biotechnology Information (NCBI) website (54). The *E. coli* quality control strain ATCC 25922 was kindly provided by the Laboratory of Pharmacology and Toxicology, College of Veterinary Medicine, Jilin Agricultural University.

### 2.2 Antimicrobial susceptibility testing

The susceptibility of the isolates to 12 antimicrobial agents consist of Kanamycin, Ofloxacin, Doxycycline, Cefotaxime, Norfloxacin, Amikacin, Cefradine, Amoxicillin, Gentamicin, Bacitracin, Cefoperazone/Sulbactam and Florfenicol was investigated according to the standards recommended by the Clinical and Laboratory Standards Institute (CLSI), using *E. coli* ATCC 25922 as the quality control strain with the Kirby-Bauer disk diffusion method. The results were interpreted according to the CLSI guidelines and the manufacturer’s reference documentation for the susceptibility disks, and were categorized as Susceptible (S), Intermediate (I), or Resistant (R) (24). Strains resistant to three or more classes of antibiotics were defined as multidrug-resistant (MDR) (25).

### 2.3 Detection of resistance genes

Genomic DNA of the isolated bacterial strains was extracted using a commercial bacterial genomic DNA extraction kit according to the manufacturer’s instructions (TransGen Biotech, Beijing, China). Primers for *E. coli* resistance genes and virulence genes were designed using Primer Premier 5.0 software, according to Wang Z (23). Universal primers for the 16S rRNA gene were synthesized by Comate Bioscience Co., Ltd. (Jilin, China) and subsequently employed in PCR detection. The primer sequences and amplification conditions are detailed in Supplementary Table 1. The presence of resistance genes in the isolates was examined using PCR. The PCR reaction system consisted of 12.5 μL of 2 × Fine Taq PCR SuperMix, 7.5 μL ddH₂O, 1 μL each of forward and reverse primers, and 3 μL DNA template.2 × Fine Taq PCR SuperMix were synthesized by Vazyme Bioscience Co., Ltd. (Jilin, China). The PCR reaction conditions were 95 °C for 5 min, 35 cycles at 94 °C for 30 s, 56 °C for 30 s, and 72 °C for 40 s, followed by 72 °C for 10 min. The PCR products were identified by 1.5% agarose gel electrophoresis.

### 2.4 Whole-genome sequencing of multidrug-resistant strains

Genomic DNA of the isolated bacterial strains was extracted using a commercial bacterial genomic DNA extraction kit according to the manufacturer’s instructions 3edd (TransGen Biotech, Beijing, China), and its concentration and quality were determined. Purity and integrity were assessed using a Qubit spectrophotometer (Invitrogen, Waltham, MA, USA) and a NanoDrop spectrophotometer (Thermo Fisher Scientific, Waltham, MA, USA). Sequencing libraries were prepared using the TruSeq DNA Sample Preparation Kit (Illumina, San Diego, CA, USA) and the Template Preparation Kit (Pacific Biosciences, Menlo Park, CA, USA). Genome sequencing was performed by Personal Biotechnology Co., Ltd. (Shanghai, China) using the Illumina NovaSeq (insert size 400 bp)sequencing platform, yielding raw sequencing data (20). Data were filtered using AdapterRemoval (Lindgreen, 2012) (58) and SOAPec (Luo et al., 2012) (59). The filtered data were assembled using SPAdes (Bankevich et al., 2012) (60) and A5-miseq (Coil et al., 2014) (61) to construct scaffold sequences and contigs (26, 28). The genomic sequences were obtained after correction using Pilon software (62).

### 2.5 Data analysis

Gene prediction was performed using GeneMarkS v4.32 software (30). The gene sequences were cross-referenced with the Gene Ontology (GO) and Kyoto Encyclopedia of Genes and Genomes (KEGG) databases for functional annotation (31, 32). Resistance genes were identified by comparing the sequences with the CARD database using ResFinder software (33, 34). Plasmid replicon types in the assembly results were identified using PlasmidFinder software (35). TransposonPSI software was used to predict transposons in the bacterial genomes (36). ISEScan software was used for the identification and annotation of full-length or partial IS elements in prokaryotic genomes (37, 38). IntegronFinder software was used to identify integrons in the bacterial genomes (39).

### 2.6 Statistical analysis

Statistical analysis was performed using the GraphPad Prism 8 software package (Graph Software, San Diego, CA, USA). All data are expressed as the mean ± standard error based on three independent experiments. A *p*-value < 0.05 was considered statistically significant.

## 3 Results

### 3.1 Identification of *E. coli*

Through bacterial isolation and identification, 50 suspected strains were isolated on *E. coli* chromogenic medium and were numbered 1–50. DNA was extracted from the isolates and amplified using 16S rRNA gene PCR, yielding specific bands of 1,369 bp. The sequencing results were compared against the NCBI database using BLAST, indicating over 99% sequence identity with *E. coli*, thereby confirming the isolates as *E. coli*.

### 3.2 Results of antimicrobial susceptibility testing

The antimicrobial susceptibility test results for the 50 *E. coli* strains are shown in Table 1. Among all strains, nine exhibited resistance rates exceeding 80%. Strains 15, 24, 27, and 36 sequenced isolates showed the highest resistance levels, with rates of 83, 83, 92, and 92%, respectively. Additionally, Strains 4, 5, 21, 31, 44, and 45 also demonstrated considerable resistance, with rates as high as 75%. Furthermore, all the strains exhibited MDR are shown in Table 2, and all were resistant to bacitracin. The rates of resistance were gentamicin 82%, amikacin 78%, ofloxacin and norfloxacin 56%, doxycycline 44%, cefotaxime 58%, amikacin and florfenicol 40%, and cefradine and amoxicillin 60%. All the strains were susceptible to cefoperazone/sulbactam (see Supplementary Table S2).

**Table 1:** Drug sensitivity test results.

Name	Number of drug-resistance strains	Drug-resistance rate (%)
Kanamycin	40/50	80
Ofloxacin	29/50	58
Doxycycline	22/50	44
Cefotaxime	29/50	58
Norfloxacin	28/50	56
Amikacin	20/50	40
Cefradine	29/50	58
Amoxicillin	30/50	60
Gentamicin	41/50	82
Bacitracin	50/50	100
Cefoperazone/Sulbactam	0/50	0
Florfenicol	22/50	44

**Table 2:** Drug sensitivity of all strains test results.

Strain No	Drug-resistance rate (%)	Strain No	Drug-resistance rate (%)
1	42	26	67
2	58	27	92
3	17	28	50
4	75	29	33
5	75	30	83
6	25	31	75
7	50	32	67
8	33	33	25
9	17	34	83
10	25	35	42
11	67	36	92
12	25	37	83
13	58	38	67
14	50	39	58
15	83	40	67
16	83	41	50
17	25	42	67
18	33	43	33
19	17	44	75
20	67	45	75
21	75	46	50
22	25	47	50
23	50	48	83
24	83	49	67
25	67	50	67

### 3.3 Detection of resistance genes

The resistance genes detected in the 50 *E. coli* isolates are shown in Table 3. The sulfonamide-resistance gene *sul2* had a detection rate of 100%, while *TEM-1,* responsible for *β*-lactam resistance, and *tetR* associated with tetracycline resistance had detection rates of 90% or above. The MDR-associated gene *QacH*, the chloramphenicol-resistance gene *floR*, the aminoglycoside-resistance gene *strB*, and the β-lactam-resistance gene *CTXM-55* had detection rates of 80% and above. These results indicate that the predominant genes involved in antimicrobial resistance in *E. coli* from diarrheic calves in the Ulagai region are *sul2*, *TEM-1*, *tetR*, *strB*, *QacH*, *floR*, and *CTXM-55*. Furthermore, the resistance phenotypes of most strains corresponded to their genotypic resistance profiles.

**Table 3:** Identified drug-resistance genes.

Drug resistance gene	Number of detected	Detection rate (%)
QacH	40	80
qnrB	4	8
sul1	20	40
tetA	39	78
TEM-1	45	90
qnrS	8	16
sul3	2	4
sul2	50	100
strB	44	88
aadA2	27	54
cmlA6	11	22
qnrD	35	70
CTXM-55	44	88
aadA5	14	28
AAC(3)-Iia	19	38
tetD	37	74
tetR	47	94
floR	40	80

### 3.4 Whole-genome sequencing of multidrug-resistant strains

#### 3.4.1 Genome assembly statistics

**Figure 1:**
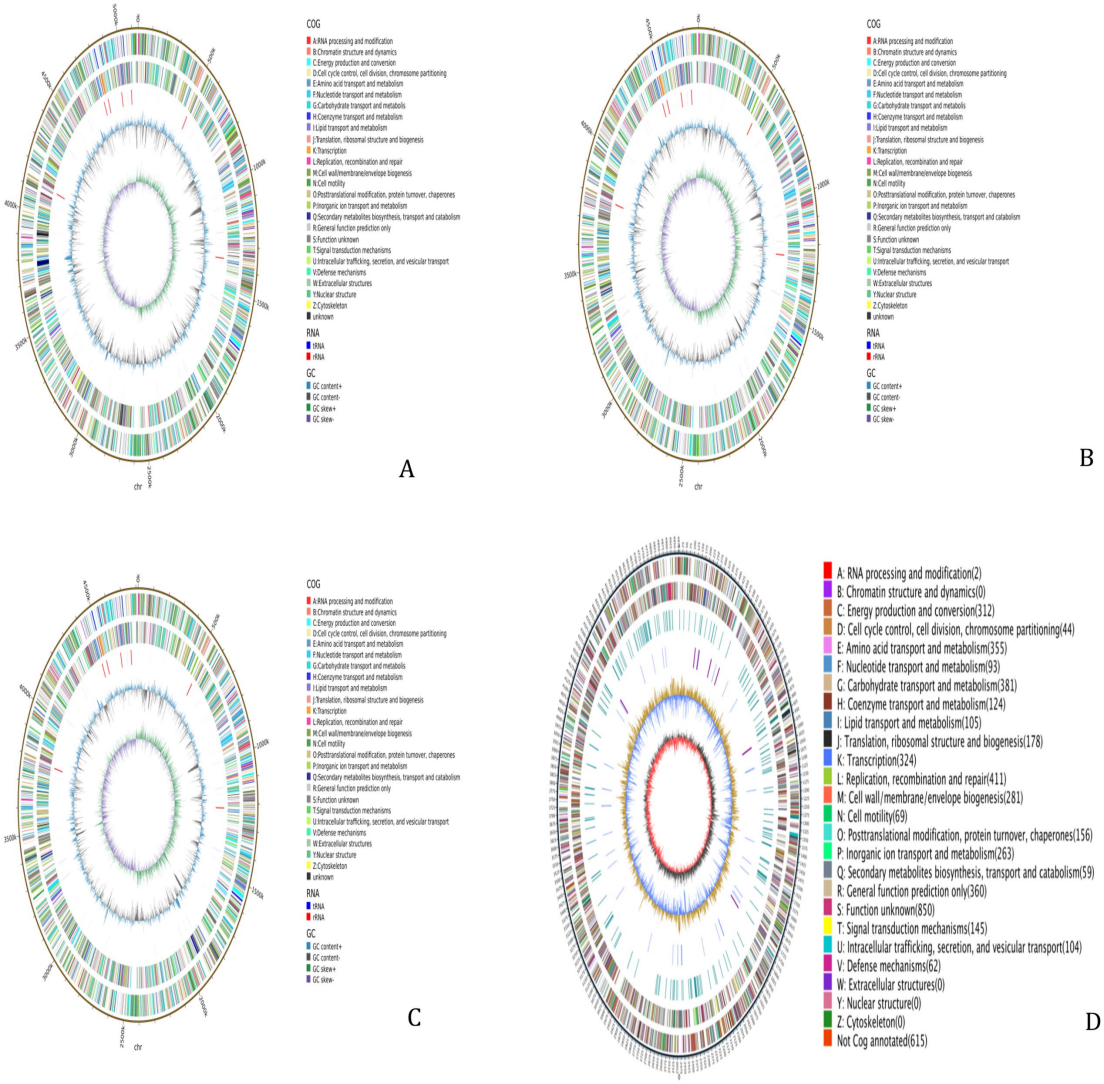
Whole-genome maps. **(A)** Whole genome map of strain 24. **(B)** Whole genome map of strain 27. **(C)** Whole genome map of strain 36. **(D)** Whole genome map of strain 15. The outermost circle is the genome size indication, each scale is 5 kb; the second and third circles are the genes on the positive and negative strands of the genome, respectively, with different colors representing different COG functional classifications; the fourth circle is the repetitive sequences; the fifth circle is the tRNAs and rRNAs, with tRNAs in blue and rRNAs in purple; the sixth circle is the GC-content, and the light yellow portion indicates that the GCcontent of the region is higher than the average GC content of the genome, the higher the peak the greater the difference with the average GC content, and the blue part indicates that the GC content of the region is lower than the average GC content of the genome; the innermost circle is the GC-skew, the dark gray represents the region where the G content is greater than the C, and the red represents the region where the C content is greater than the G.

The WGS results for the 4 MDR strains revealed that Strain 24 had a total genome length of 5,144,828 bp, containing five plasmids, with a G + C content of 50.61%. Strain 27 had a total genome length of 4,798,224 bp, containing three plasmids, with a G + C content of 50.66%. The total genome length of Strain 36 was 4,813,249 bp, with six plasmids and a G + C content of 50.65%, while the length of the Strain 15 genome was 5,450,201 bp with two plasmids and a G + C content of 50.65%. The sequences have been deposited in the NCBI Sequence Read Archive (SRA) under the accession numbers CP195580-CP195584, CP195607-CP195610, CP195776-CP195782, and CP195330-CP195332 for Strains 24, 27, 36, and 15, respectively, and are publicly accessible. Circular whole genome maps of the four *E. coli* strains are shown in Figure 1. Comparative analysis of the four genome maps revealed that Strain 15 exhibited advantages in amino acid transport and metabolism, DNA replication/recombination/repair, carbohydrate transport and metabolism, and lipid transport and metabolism compared to the other three strains, but was less efficient in the biosynthesis, transport, and catabolism of secondary metabolites.

#### 3.4.2 KEGG and GO functional annotation

KEGG pathway annotation was performed on the gene sequences of the four *E. coli* strains. The results showed that the annotated genes were primarily involved in metabolic pathways, including amino acid synthesis and carbon metabolism. GO functional annotation indicated that most genes were most enriched in the molecular function category, particularly in RNA binding, which is closely related to the life activities of the strains. Compared to the other three strains, the genes in Strain 15 showed greater enrichment in metabolic functions (see Supplementary Figures S1A–D).

#### 3.4.3 Analysis of resistance genes

The prediction of resistance genes in the four *E. coli* strains 24, 27, 36, and 15 carried 152, 166, 162, and 50 resistance genes, respectively. Among these, 135, 148, 137, and 49 resistance genes were associated with the chromosomal genes, respectively, and 17, 18, 25, and 1 with the plasmid genomes. The total number of distinct resistance gene types in the strains were 26, 28, 29, and 26, respectively (see Supplementary Tables S3–S6). The resistance genes in the chromosomal genomes of the four strains mediated resistance to fluoroquinolones, tetracyclines, aminoglycosides, *β*-lactams, and macrolides, among others, while the genes in the plasmid genomes mediated resistance to sulfonamides, quinolones, β-lactams, aminoglycosides, and fosfomycin, among others. The mechanisms associated with resistance to these antibiotics primarily included antibiotic efflux, modification-induced inactivation, target replacement, and alteration of antibiotic targets.

#### 3.4.4 Analysis of virulence genes

The prediction of virulence genes in the four *E. coli* strains 24, 27, 36, and 15 carried 314, 253, 263, and 988 virulence genes, respectively. Among these, 299, 236, 246, and 988 virulence genes were associated with the chromosomal genes, respectively, and 15, 17, 17 and 27 with the plasmid genomes. The virulence genes in the chromosomal genomes of the four strains mediated resistance to Immune modulation, Nutritional/Metabolic factor, Motility, Effector delivery system, and Adherence, among others, while the genes in the plasmid genomes mediated resistance to Adherence, Nutritional/Metabolic factor.

#### 3.4.5 Multilocus sequence typing (MLST) analysis

MLST analysis identified three sequence types (STs): ST69, ST744, and ST392, with ST744 being the predominant type. Plasmid typing revealed that the four *E. coli* isolates collectively carried seven plasmid types are shown in Table 4, among which IncF and IncI plasmids were the most prevalent. The IncF-type plasmid was present in all isolates, primarily belonging to the IncFIB and IncFII subtypes.

**Table 4:** Plasmid types.

SeqID	Database	Plasmid	Identity
plasmid1(E24)	enterobacteriales	IncFIB(AP001918)	98.68
plasmid1(E24)	enterobacteriales	IncFII (29)	100
plasmid1(E24)	enterobacteriales	IncFII(pCoo)	96.18
plasmid2(E24)	enterobacteriales	IncHI2	99.69
plasmid2(E24)	enterobacteriales	IncHI2A	99.52
plasmid3(E24)	enterobacteriales	IncY	99.61
plasmid4(E24)	enterobacteriales	IncI1-I(Alpha)	100
plasmid5(E24)	enterobacteriales	Col(pHAD28)	92.25
plasmid1(E27)	enterobacteriales	IncFIB(AP001918)	98.39
plasmid1(E27)	enterobacteriales	IncFIC(FII)	95.79
plasmid2(E27)	enterobacteriales	IncI1-I(Alpha)	100.00
plasmid3(E27)	enterobacteriales	IncN	99.81
plasmid1(E36)	enterobacteriales	IncFIB(AP001918)	98.39
plasmid1(E36)	enterobacteriales	IncFIC(FII)	95.79
plasmid1(E36)	enterobacteriales	IncN	100.0
plasmid2(E36)	enterobacteriales	IncFII(pHN7A8)	100.0
plasmid2(E36)	enterobacteriales	IncN	100.0
plasmid2(E36)	enterobacteriales	IncR	100.0
plasmid3(E36)	enterobacteriales	IncI1-I(Alpha)	99.3
plasmid4(E36)	–	–	–
plasmid5(E36)	–	–	–
Plasmid6(E36)	enterobacteriales	Col440I	95.45
plasmid1(E15)	-	-	-
plasmid2(E15)	enterobacteriales	IncFIA(HI1)	95.08
plasmid2(E15)	enterobacteriales	IncFIB(AP001918)	99.27
Plasmid2(E15)	enterobacteriales	IncFII	95.83
Plasmid3(E15)	enterobacteriales	IncX1	98.66

#### 3.4.6 Analysis of mobile elements

**Figure 2:**
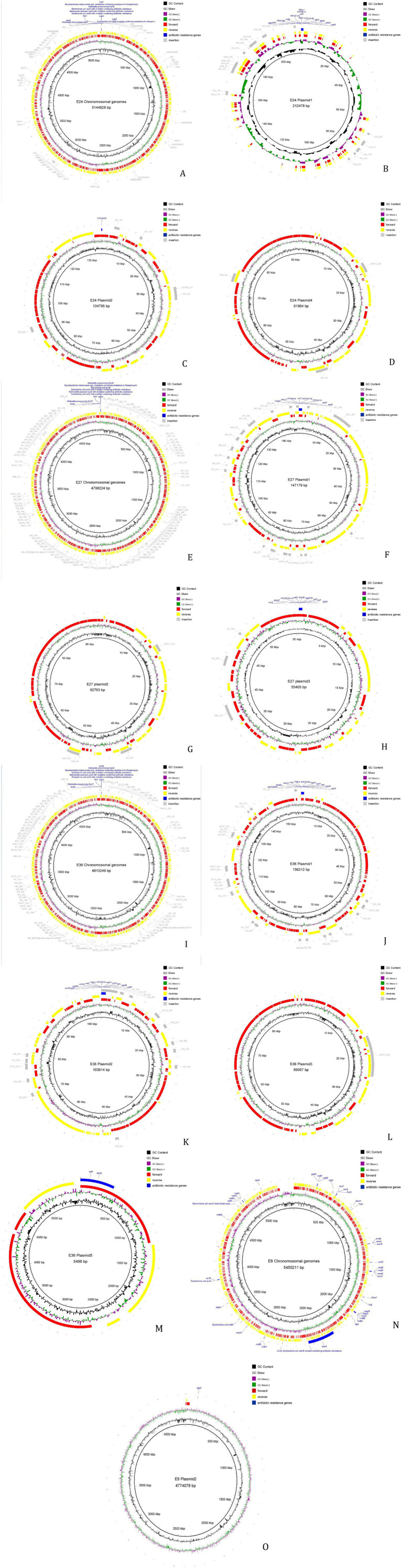
Analysis result of mobile components. **(A)** Result of mobile components analysis of strain 24 Chromosomal genomes. **(B)** Result of mobile components analysis of strain 24 plasmid 1. **(C)** Result of mobile components analysis of strain 24 plasmid 2. **(D)** Result of mobile components analysis of strain 24 plasmid 4. **(E)** Result of mobile components analysis of strain 27 Chromosomal genomes. **(F)** Result of mobile components analysis of strain 27 plasmid 1. **(G)** Result of mobile components analysis of strain 27 plasmid 2. **(H)** Result of mobile components analysis of strain 27 plasmid 3. **(I)** Result of mobile components analysis of strain 36 Chromosomal genomes. **(J)** Result of mobile components analysis of strain 36 plasmid 1. **(K)** Result of mobile components analysis of strain 36 plasmid 2. **(L)** Result of mobile components analysis of strain 36 plasmid 3. **(M)** Result of mobile components analysis of strain 36 plasmid 5. **(N)** Result of mobile components analysis of strain 15 Chromosomal genomes. **(O)** Result of mobile components analysis of strain 15 plasmid 2.

The MGE in the genomes were predicted and analyzed using bioinformatics. The results showed that the MGEs in all four strains were primarily IS, including a large number of repetitive sequences (Figure 2). Complete integrons were found on plasmid 3 of strain 27 and on plasmids 1 and 2 of strain 36. The MGEs involved in the transmission of resistance genes were mostly insertion sequences, with most belonging to the IS6 family. Horizontal transfer of these IS elements can regulate the expression of resistance genes or assist in their horizontal transfer. Insertion sequences involved in the horizontal transfer of resistance genes were found on both the chromosome and plasmids 1, 2, and 4 of strain 24, on the chromosome and plasmids of strain 27, and on the chromosome and plasmids 1 and 2 of strain 36. However, none of the four strains contained composite transposons (see Supplementary Tables S7–S9).

## 4 Discussion

Diarrhea in calves is common in the global cattle industry, resulting in significant economic losses and severely hindering the development of cattle farming (8, 40). A relatively high incidence of calf diarrhea has been reported in Northeast China, with enterotoxigenic *E. coli* (ETEC) being a major causative agent. Antibiotics, the first-choice drugs for preventing and treating animal diseases, play a crucial role in the treatment of bacterial diseases. However, extensive long-term antibiotic use has led to the development of increasingly severe bacterial resistance, extension of the resistance spectra, and the frequent emergence of MDR strains. The persistent increase in *E. coli* resistance has become a growing public health safety concern (41–47).

In this study, antimicrobial susceptibility testing of 50 *E. coli* strains isolated from diarrheic calves in the Ulagai region indicated varying degrees of resistance to 12 antibiotics. All isolates were resistant to bacitracin, this is due to the fact that the drug has been broadly administered in this area, over 80% were resistant to gentamicin, and all exhibited MDR, although they remained susceptible to cefoperazone/sulbactam. In recent domestic studies, Zhang et al. (44) reported high levels of antibiotic resistance genes, including *gyrB*, *blaTEM*, *floR*, *tetD*, *gyrA*, *catA1*, and *tetB*, in 1685 diarrheic calves, with the quinolone resistance gene *gyrB* and the *β*-lactam resistance gene *blaTEM* detected at 100%. Wang et al. (23) analyzed MDR *E. coli* from diarrheic calves in the Tongliao region and observed high levels of resistance, with resistance rates to sulfadiazine sodium, enrofloxacin, and ciprofloxacin of up to 100%. The resistance genes *TEM-1*, *TEM-206*, *strA*, *strB*, *qacH*, and *blaCTX* were all found to be 100%. Additionally, Yan et al. (45) found highly resistant pathogenic *E. coli* in fecal samples from diarrheic calves on farms around Hohhot, with resistance rates to penicillin and ampicillin of 100%, and over 50% against cephalosporins. In international studies, Srinivasan et al. (46) reported that all of their 135 bovine *E. coli* isolates from New York were MDR, with resistance rates to tetracycline, sulfisoxazole, streptomycin, aztreonam, and ampicillin of 24.8, 34.1, 40.3, 97.7, and 98.4%, respectively, although they were susceptible to cinoxacin and ciprofloxacin. Furthermore, Eldesoukey et al. (47) found that all EPEC isolates from rectal swabs of diarrheic dairy cows in Egypt were resistant, with rates of resistance to ampicillin, tetracycline, cefazolin, and ciprofloxacin of 100, 89.3, 71, and 64.3%, respectively. A compilation of these findings suggests that the differences in *E. coli* resistance profiles to various antimicrobials across species and regions. Moreover, we observed a high overlap between the resistance genes detected in the present study and those reported from diarrheic calves in the Tongliao region. In subsequent research, we will continue to monitor the resistance profiles of *E. coli* strains isolated from diarrheic calves in Ulagai and compare them with those from the Tongliao region.

Through virulence gene detection, the most prominent findings in our data were the ten virulence gene pairs with a 100% detection rate: *iroN*, *ompT*, *hlyF*, *Iss-F1*, *phoA*, *luxS*, *pfs*, *fimC*, *iucD*, and *ompA*. Additionally, three genes—*iutA*, *Irp2*, and *Iss-F2*—had detection rates exceeding 90%. Among the remaining genes, *fyuA* and *hlyE* were not detected (0% detection rate). These data provide valuable support for the investigation of *E. coli* virulence genes in diarrheic calves in the Ulagai region. A study by Uruguayan scholars examining 21 *E. coli* virulence genes in fecal samples from 252 dairy calves found that the *iucU*, *f17A*, *afa8E*, *papC*, *clpG*, and *f17G(II)* genes were the most prevalent, with detection rates of 81.3, 48.4, 37.3, 35.7, 34.1, and 31.3%, respectively (56). Their results indicated high detection rates for fimbrial adhesins, which is consistent with previously mentioned studies and the findings of the present research. Korean researchers (57), through detection of *E. coli* virulence genes in pre-weaned calf feces, concluded that the incidence of *E. coli* is age-related, but found no association between *E. coli* pathogenic genes and calf age or diarrhea. This may suggest that the mechanism of calf diarrhea related to *E. coli* is not primarily reflected in the existing virulence genes, and the underlying mechanisms may be more complex. Iranian scholar Reza Ghanbarpour, upon analyzing pathogenic genes in *E. coli* isolated from dairy cattle, found that among the isolates, 11.81% carried *iucD*, 9.44% possessed *f17c-A*, 9.44% had *cnf2*, 7.87% contained *f17b-A*, 6.29% had *afaD-8* and *afaE-8*, 3.14% carried *f17d-A*, 0.78% had *cnf1*, and 0.78% possessed *clpG*. With the exception of the *clpG* and *f17d-A* genes, which were found alone, all other detected pathogenic genes existed in combinations with other genes. No isolates contained genes for *F17a-A*, adhesins, P or S fimbriae (58). Thus, from a global perspective, the detection rates of adhesin-like virulence genes are not consistent, and actual detection results still vary across different regions.n the study by Shi et al. (55), it was demonstrated that the diversity and abundance of antibiotic resistance genes (ARGs) in diarrheic calves were significantly higher than those in healthy calves. In the present study, the diversity and abundance of annotated antibiotic resistance genes were also higher in the diarrheic group compared to the healthy group, further validating the complex relationship between diarrheic behavior in calves and the presence of antibiotic resistance genes in their microbiota. In the functional annotation results of virulence factors from the VFDB in this study, eight of the top ten virulence genes—namely *fdeC*, *entF*, *espX4*, *ompA*, *entE*, *entD*, *kdsA*, and *fimA*—are closely associated with *E. coli*. These genes may be present in many bacteria, particularly *E. coli*, which aligns with the findings of Shi et al. (55). This undoubtedly provides strong evidence supporting the notion that virulence genes in the gut microbiota of diarrheic calves may be predominantly dominated by *E. coli*.

Cefoperazone/sulbactam is one of the widely used clinical treatments for Gram-negative bacterial infections. However, due to its long-term use and the increasing prevalence of multidrug resistance, its antibacterial efficacy has significantly declined. Currently, polymyxin B—a member of the colistin class—has become a last-line monotherapy for infections caused by resistant strains (48, 49). MDR in *E. coli* is associated with its resistance phenotype. The detection of resistance genes revealed high prevalence and detection rates for genes related to *β*-lactam, aminoglycoside, fosfomycin, and fluoroquinolone resistance. Overall, the results of this study indicate a serious antimicrobial resistance problem in *E. coli* within the Ulagai region, which appears to be worsening over time. Therefore, optimization of local measures for preventing and controlling resistance is urgently needed.

Whole-genome sequencing (WGS) enables in-depth analysis of the genetic information of resistant strains (50). In this study, WGS of four MDR *E. coli* strains revealed that strains 24, 27, 36, and 15 carried five, three, six, and two plasmids, respectively. GO functional annotation of the four *E. coli* strains indicated significant enrichment of ARGs in all three GO categories of cellular components, metabolic processes, and molecular functions. A greater number of genes in Strain 24, in particular, were involved in metabolic activities, cell membrane and cytoplasmic components, and transport functions, suggesting the importance of these processes in antibiotic efflux and the prevention of uptake. The KEGG analysis showed significant enrichment of the ARGs in pathways associated with signal transduction, the processing of genetic information, amino acid metabolism, and carbon metabolism, consistent with the GO annotation results. Studies have shown that *E. coli* can develop resistance through various mechanisms, especially those associated with efflux pumps, enzymatic modification, biofilm formation, and altered cell membrane permeability. Predictions using the CARD database showed that all four *E. coli* strains in this study exhibited MDR, which was primarily linked to antibiotic efflux. Strain 36 carried more ARGs on its plasmids than the other three strains, and testing of itsantimicrobial susceptibility confirmed resistance to multiple antibiotics.

MGEs serve as critical vectors for the dissemination of AMR in bacteria. They facilitate the horizontal transfer of ARGs through various mechanisms, significantly accelerating the evolution and spread of multidrug-resistant strains (51). In this study, IncF and IncI plasmids were identified as the most prevalent types among the four bacterial isolates subjected to whole-genome sequencing. IncF plasmids are highly common in Enterobacteriaceae, particularly in *E. coli*, and play an essential role in mediating the transmission of *blaCTX-M*. IncI plasmids are currently the most frequently reported vectors carrying the *mcr-1* gene. As self-transmissible conjugative plasmids, both IncF and IncI types are capable of independent conjugative transfer and can also co-transfer, thereby enhancing the flexibility and efficiency of horizontal gene transfer and further promoting the dissemination of resistance genes. Furthermore, the prediction of gene islands identified numerous IS6 family insertion sequences. IS enrichment may indicate that the gene island was recently acquired through exogenous DNA capture (e.g., via phage, plasmid, or conjugative transposons). IS elements encode transposases that can mediate the cleavage, recombination, and integration of gene islands, promoting their HGT within the genome or between strains (52). Integron prediction identified complete integrons on plasmid 3 of strain 27 and on plasmids 1 and 2 of strain 36. An integron is a mobile DNA molecule that, through association with transposons or conjugative plasmids, enables the horizontal spread of MDR genes among bacteria (53). The prediction of these mobile elements indicates a risk of horizontal transfer for the ARGs carried by the isolated strains. But because of the sample size of strains in our study is relatively insufficient. Therefore, continuous strengthening of the monitoring of *E. coli* ARGs from diarrheic calves is essential to effectively prevent future public health risks.

## 5 Conclusion

This study isolated, identified, and analyzed antimicrobial resistance in *E. coli* from fecal samples from diarrheic calves in the Ulagai region of China. The findings revealed high levels of antibiotic resistance in the local *E. coli* population. The resistance rate to bacitracin was 100%, and the detection rate of the ARG *sul2* was also 100%. Whole-genome sequencing demonstrated that the four sequenced MDR strains carried plasmids harboring ARGs, and these resistance genes were abundant within insertion sequences. Furthermore, the plasmids of strains 27 and 36 contained complete integrons. The presence of these mobile elements increases the risk of ARG transmission between bacteria, leading to a significant increase in strain resistance.

## Data Availability

The datasets presents in this study can be found in online repositories. The names of the repository/repositories and accession number(s) can be found at: https://www.ncbi.nlm.nih.gov/genbank/, CP195580-CP195584, CP195607-CP195610, CP195776- CP195782, and CP195330-CP195332.
