# Supplementary figures and images for: Research of antimicrobial resistance and its associated genes distribution in Escherichia coli from diarrheic calves in the Ulagai region of China
Source: Front Vet Sci. 2025 Nov 24;12:1685829. doi: 10.3389/fvets.2025.1685829 (PMC12683719; doi:10.3389/fvets.2025.1685829)

## Slide 1
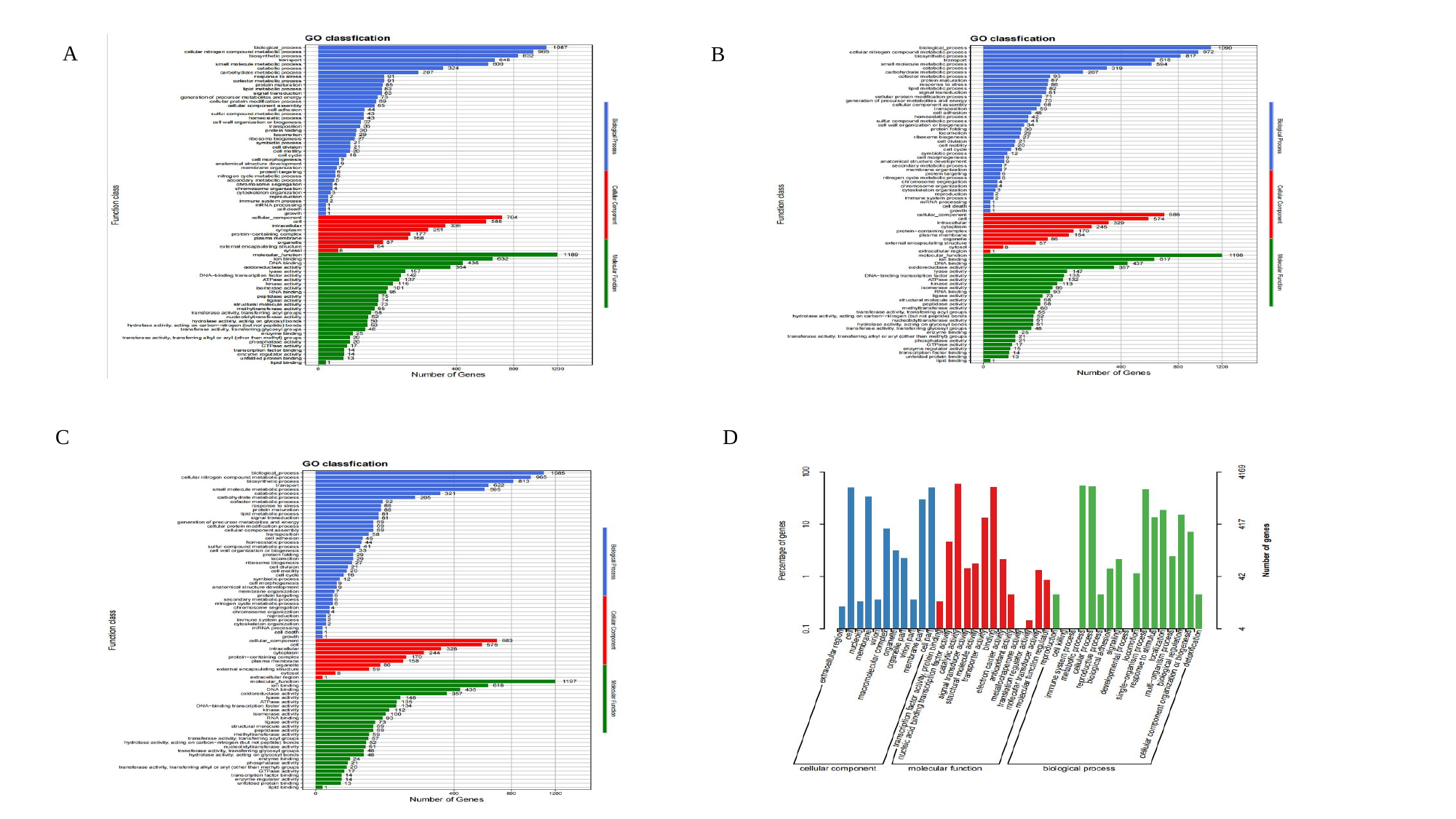

A
B
C
D

## Slide 2
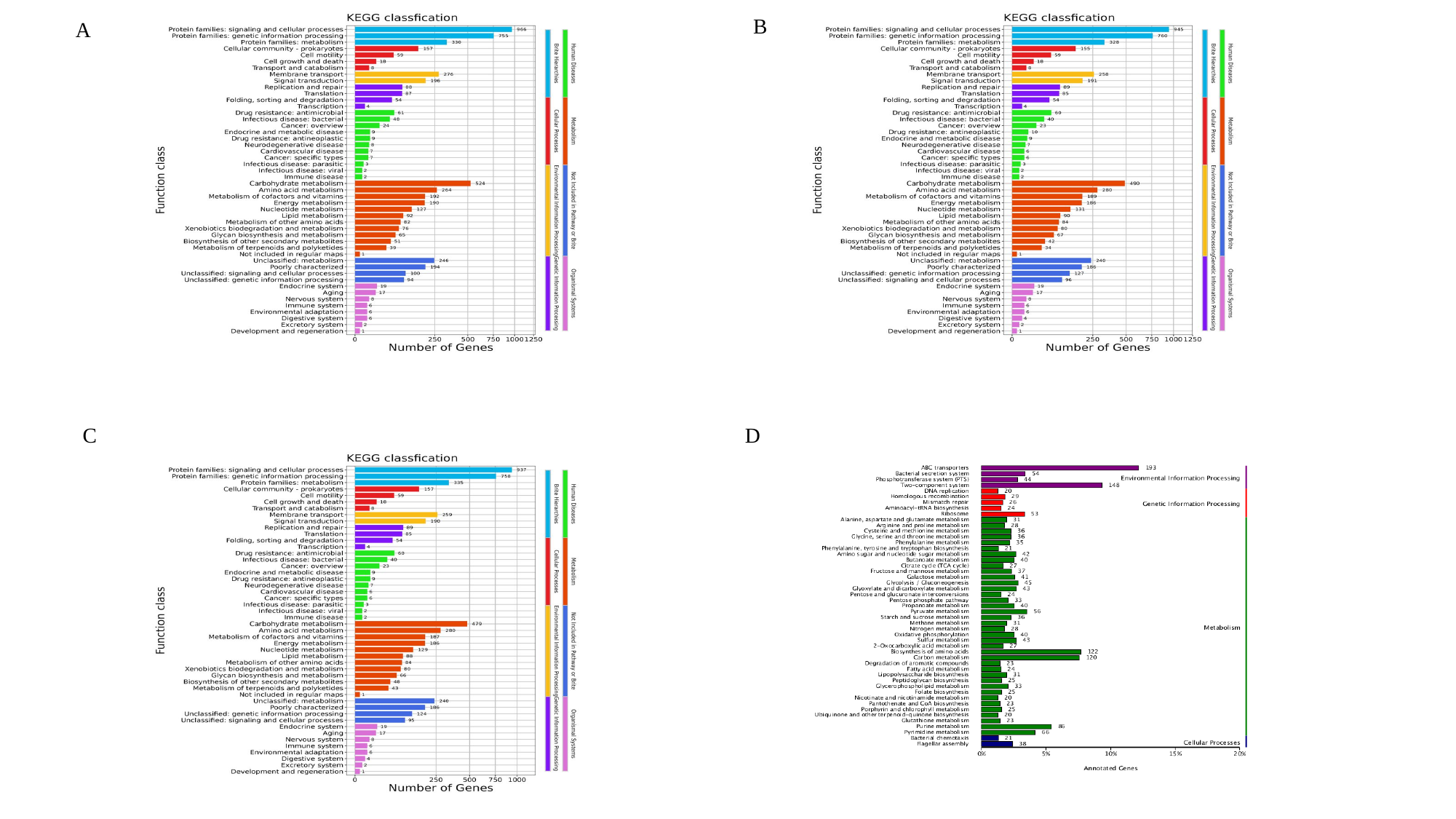

B
A
C
D

Supplement: Supplementary file 1 [file Presentation_1.pptx]
