# Supplementary material for: Research of antimicrobial resistance and its associated genes distribution in Escherichia coli from diarrheic calves in the Ulagai region of China
Source: Front Vet Sci. 2025 Nov 24;12:1685829. doi: 10.3389/fvets.2025.1685829 (PMC12683719; doi:10.3389/fvets.2025.1685829)
Supplement: Supplementary file 2 [file Table_1.docx]

Table S1 Primer sequences of drug-resistance genes

| Primer name | Primer sequence (5´→3´) | The length of the amplification/bp |  | Primer name | Primer sequence (5´→3´) | The length of the amplification/bp |
| --- | --- | --- | --- | --- | --- | --- |
| aPhA1 | F:AAAGCCGTTTCTGTAATGAAGGAG  R:GGCAATCAGGTGCGACAATCT | 642 |  | tetD | F: ACAGCATTCTCGCTATCA  R: TATTCGCCACATCATCAA | 146 |
| strB | F:GCGTTGCTCCTCTTCTCCAT  R: ACCTTTTCCAGCCTCGTTTG | 723 |  | tetR | F: AACAACCCGTAAACTCGC  R: GGTGCCTATCTAACATCTCAAT | 116 |
| strA | F: ATCGCAGATAGAAGGCAAGGC  R: AACTGGCAGGAGGAACAGGA | 574 |  | sul1 | F: TTTCCTGACCCTGCGCTCTAT  R: GTGCGGACGTAGTCAGCGCCA | 425 |
| aadA25 | F: ACTATCAGAGGTGCTAAGCGTCAT  R: CACGTAGTGAACAAATTCTTCCAAC | 724 |  | sul2 | F: CCTGTTTCGTCCGACACAGA  R: GAAGCGCAGCCGCAATTCAT | 435 |
| aadA17 | F:ATTCTTGCGGGTATCTTCG  R:CTGGGCAGGTAGGCGTTT | 309 |  | QacH | F: TCGCACTCAAGTCCATCC  R: CGACGCCACTAACGATAAG | 150 |
| aadA5 | F:CTTCAGTTCGGTGAGTGGC  R:AGGTCGTCGTCCTCGCTAC | 398 |  | Int1 | F: CCTCCCGCACGATGATC  R: TCCACGCATCGTCAGGC | 280 |
| sul3 | F:ATGAGCAAGATTTTTGGAATCGTAA  R:CTAACCCTAGGGCTTTGGATATTT | 792 |  | catl | F: CAATGAAAGACGGTGAGC  R: ACAAACGGCATGATGAAC | 362 |
| TEM-1 | F:TTTTCGTGTCGCCCTTAT  R:GCTCGTCGTTTGGTATGG | 512 |  | cmlA6 | F: GTACGACAGCGAGCACAA  R: CCACCTCCCAGTAGAACG | 124 |
| TEM-206 | F:TGAATGAAGCCATACCAA  R:AGATAACTACGATACGGGAG | 279 |  | floR | F: CTGAACACGACGCCCGCTAT  R: GGACCGCTCCGCAAACAA | 751 |
| blaCTX | F:CGCTTTGCGATGTGCAG  R:ACCGCGATATCGTTGGT | 550 |  | mPrF | F: GGATGCAGGTTTGGTCTG  R: TCGTCAATACGGTTCTCACT | 282 |
| CTXM-55 | F:GCTGGGTAAAGCATTGGG  R:TAGGTTGAGGCTGGGTGA | 224 |  | qnrS | F: ACGACATTCGTCAACTGCAA  R: TAAATTGGCACCCTGTAGGC | 417 |
| AAC(3)-Iia | F:TCACGAACTCGGTCACGC  R:CCAAGCATCGGCATCTCA | 177 |  | blaSHV | F: GGGTTATTCTTATTTGTCGC  R: TTAGCGTTGCCAGTGCTC | 567 |
| aadA2 | F: CATCCCGTGGCGTTATCC  R: CTGGGCAGGTAGGCGTTT | 370 |  | qnrB | F: GATCGTGAAAGCCAGAAAGG  R: ACGATGCCTGGTAGTTGTCC | 469 |
| aadA | F: ATCTGGCTATCTTGCTGACA  R: TTGGTGATCTCGCCTTTC | 388 |  | qnrC | F:ATTTCTCACAGGCAAACT  R: CTGGAATAACAATCACCC | 666 |
| tetA | F: GCTACATCCTGCTTGCCTTC  R: CATAGATCGCCGTGAAGAGG | 210 |  | qnrD | F:TTTTCGCTAACTAACTCGC  R:GAAAGGATAAACAGGCAAAT | 984 |
| 16S rRNA | F: AGAGTTTGATCCTGGCTCAG  R: GGTTACCTTGTTACGACTT | 1369 |  |  |  |  |

Table S2 Drug sensitivity test results

| Strain No | Kanamycin | Ofloxacin | Doxycycline | Cefotaxime | Norfloxacin | Amikacin | Cefradine | Amoxicillin | Gentamicin | Bacitracin | Cefoperazone/Sulbactam | Florfenicol |
| --- | --- | --- | --- | --- | --- | --- | --- | --- | --- | --- | --- | --- |
| 1 | S（16） | R（0） | I（10） | S（32） | R（0） | S（20） | S（22） | R（0） | R（0） | R（0） | S（22） | S（24） |
| 2 | S（18） | R（0） | R（0） | R（0） | S（16） | S（22） | R（0） | R（0） | R（0） | R（0） | S（20） | S（28） |
| 3 | S（16） | S（18） | I（10） | S（28） | S（20） | S（20） | S（22） | R（0） | S（16） | R（0） | S（22） | S（22） |
| 4 | R（0） | R（0） | I（10） | R（0） | R（0） | R（0） | R（0） | R（0） | R（0） | R（0） | S（20） | S（22） |
| 5 | R（0） | R（0） | I（10） | R（0） | R（0） | R（0） | R（0） | R（0） | R（0） | R（0） | S（18） | S（22） |
| 6 | S（16） | S（28） | S（18） | S（30） | S（25） | S（16） | S（14） | R（0） | R（0） | R（0） | S（22） | S（24） |
| 7 | R（0） | S（16） | I（10） | R（0） | S（18） | S（20） | R（0） | S（18） | R（0） | R（0） | S（20） | R（8） |
| 8 | S（16） | R（0） | I（10） | S（26） | R（0） | S（16） | S（18） | R（0） | S（12） | R（0） | S（20） | S（20） |
| 9 | R（0） | S（20） | S（16） | S（26） | S（26） | I（14） | S（20） | S（24） | S（12） | R（0） | S（22） | S（26） |
| 10 | R（0） | S（20） | S（14） | S（24） | S（20） | S（16） | S（12） | S（20） | R（0） | R（0） | S（24） | S（24） |
| 11 | S（16） | R（0） | R（0） | R（0） | R（0） | I（12） | R（0） | R（0） | S（12） | R（0） | S（16） | R（0） |
| 12 | R（0） | S（20） | S（16） | S（20） | S（20） | I（14） | S（16） | S（18） | R（0） | R（0） | S（16） | S（22） |
| 13 | R（0） | R（0） | R（0） | S（20） | I（12） | I（14） | S（16） | R（0） | R（0） | R（0） | S（16） | R（0） |
| 14 | I（8） | R（0） | R（0） | S（22） | I（12） | S（16） | S（16） | R（0） | R（0） | R（0） | S（16） | R（0） |
| 15 | R（0） | R（0） | R（8） | R（0） | R（0） | I（12） | R（0） | R（0） | R（0） | R（0） | S（16） | R（0） |
| 16 | R（0） | R（0） | R（8） | R（0） | R（0） | I（12） | R（0） | R（0） | R（0） | R（0） | S（16） | R（0） |
| 17 | R（0） | S（18） | I（12） | S（20） | S（22） | I（12） | S（16） | S（24） | S（12） | R（0） | S（20） | R（0） |
| 18 | R（0） | S（20） | I（12） | S（20） | S（20） | I（12） | S（16） | S（24） | R（0） | R（0） | S（22） | R（8） |
| 19 | R（0） | S（18） | I（12） | S（24） | S（16） | I（12） | S（16） | S（22） | S（12） | R（0） | S（20） | S（20） |
| 20 | R（0） | R（0） | I（10） | R（0） | R（0） | I（12） | R（0） | R（0） | R（0） | R（0） | S（18） | S（22） |
| 21 | R（0） | R（0） | R（8） | R（0） | R（0） | I（12） | R（0） | R（0） | R（0） | R（0） | S（18） | S（20） |
| 22 | R（0） | S（24） | S（14） | S（28） | S（24） | I（14） | S（16） | S（22） | R（0） | R（0） | S（23） | S（20） |
| 23 | R（0） | S（22） | S（16） | S（24） | R（0） | R（0） | R（0） | S（16） | R（0） | R（0） | S（18） | S（16） |
| 24 | R（0） | R（0） | R（0） | R（0） | R（0） | R（0） | R（0） | R（0） | R（0） | R（0） | S（16） | S（18） |
| 25 | R（0） | R（0） | R（0） | S（22） | R（0） | R（0） | S（14） | R（0） | R（0） | R（0） | S（20） | S（14） |
| 26 | R（0） | R（0） | R（0） | S（22） | R（0） | R（0） | S（16） | R（0） | R（0） | R（0） | S（20） | S（20） |
| 27 | R（0） | R（0） | R（0） | R（0） | R（0） | R（0） | R（0） | R（0） | R（6） | R（0） | S（18） | R（0） |
| 28 | R（0） | S（18） | R（0） | S（20） | S（20） | R（0） | R（0） | S（22） | R（0） | R（0） | S（21） | S（18） |
| 29 | R（0） | S（20） | S（20） | S（24） | S（20） | R（0） | S（18） | S（24） | R（0） | R（0） | S（18） | S（16） |
| 30 | R（0） | R（0） | I（10） | R（0） | R（0） | R（0） | R（0） | R（0） | R（0） | R（0） | S（16） | R（0） |
| 31 | R（0） | R（0） | I（10） | R（0） | R（0） | I（14） | R（0） | R（0） | R（6） | R（0） | S（18） | R（0） |
| 32 | R（0） | R（0） | I（10） | R（0） | R（0） | I（14） | R（0） | R（0） | R（0） | R（0） | S（20） | S（18） |
| 33 | R（0） | S（20） | S（24） | S（24） | S（20） | I（14） | S（14） | S（16） | R（0） | R（0） | S（19） | S（23） |
| 34 | R（0） | R（0） | S（14） | R（0） | R（0） | R（0） | R（0） | R（0） | R（0） | R（0） | S（16） | R（0） |
| 35 | R（0） | S（20） | S（18） | R（0） | S（18） | I（14） | S（16） | S（16） | R（0） | R（0） | S（20） | R（0） |
| 36 | R（0） | R（0） | R（8） | R（0） | R（0） | R（0） | R（0） | R（0） | R（0） | R（0） | S（19） | R（0） |
| 37 | R（0） | R（0） | R（0） | R（0） | R（0） | I（14） | R（0） | R（0） | R（0） | R（0） | S（19） | R（9） |
| 38 | R（0） | S（20） | I（10） | R（0） | S（18） | R（0） | R（0） | R（0） | R（0） | R（0） | S（20） | R（0） |
| 39 | R（0） | S（20） | R（0） | R（0） | S（20） | R（0） | R（0） | S（16） | S（12） | R（0） | S（18） | R（9） |
| 40 | R（0） | R（0） | I（12） | R（0） | R（0） | I（14） | R（0） | S（14） | R（0） | R（0） | S（20） | R（0） |
| 41 | I（8） | S（22） | R（0） | R（0） | S（24） | R（0） | R（0） | R（0） | S（12） | R（0） | S（21） | S（20） |
| 42 | R（0） | R（0） | R（0） | R（0） | R（0） | I（14） | R（0） | R（0） | R（0） | R（0） | S（18） | R（0） |
| 43 | R（0） | S（18） | I（12） | S（20） | S（16） | R（0） | S（24） | S（24） | R（0） | R（0） | S（18） | S（20） |
| 44 | S（16） | R（0） | R（0） | R（0） | R（0） | R（0） | R（0） | S（16） | R（0） | R（0） | S（21） | R（8） |
| 45 | R（0） | R（0） | R（8） | R（0） | R（0） | R（0） | R（0） | R（0） | S（12） | R（0） | S（20） | R（8） |
| 46 | S（16） | S（20） | R（0） | R（0） | S（20） | I（14） | R（0） | R（0） | R（0） | R（0） | S（20） | S（24） |
| 47 | R（0） | R（0） | S（14） | S（20） | R（0） | I（14） | S（16） | R（0） | R（0） | R（0） | S（19） | S（22） |
| 48 | R（0） | R（0） | R（0） | R（0） | R（0） | R（0） | R（0） | S（14） | R（0） | R（0） | S（20） | R（0） |
| 49 | R（0） | S（20） | R（10） | R（0） | R（0） | R（0） | R（0） | S（20） | R（0） | R（0） | S（20） | S（22） |
| 50 | R（0） | R（0） | S（14） | R（0） | R（0） | R（0） | R（0） | S（18） | R（0） | R（0） | S（20） | S（22） |

Table S2 Primer sequences of drug-resistance genes

| Primer name | Primer sequence (5´→3´) | The length of the amplification/bp |  | Primer name | Primer sequence (5´→3´) | The length of the amplification/bp |
| --- | --- | --- | --- | --- | --- | --- |
| aPhA1 | F:AAAGCCGTTTCTGTAATGAAGGAG  R:GGCAATCAGGTGCGACAATCT | 642 |  | tetD | F: ACAGCATTCTCGCTATCA  R: TATTCGCCACATCATCAA | 146 |
| strB | F:GCGTTGCTCCTCTTCTCCAT  R: ACCTTTTCCAGCCTCGTTTG | 723 |  | tetR | F: AACAACCCGTAAACTCGC  R: GGTGCCTATCTAACATCTCAAT | 116 |
| strA | F: ATCGCAGATAGAAGGCAAGGC  R: AACTGGCAGGAGGAACAGGA | 574 |  | sul1 | F: TTTCCTGACCCTGCGCTCTAT  R: GTGCGGACGTAGTCAGCGCCA | 425 |
| aadA25 | F: ACTATCAGAGGTGCTAAGCGTCAT  R: CACGTAGTGAACAAATTCTTCCAAC | 724 |  | sul2 | F: CCTGTTTCGTCCGACACAGA  R: GAAGCGCAGCCGCAATTCAT | 435 |
| aadA17 | F:ATTCTTGCGGGTATCTTCG  R:CTGGGCAGGTAGGCGTTT | 309 |  | QacH | F: TCGCACTCAAGTCCATCC  R: CGACGCCACTAACGATAAG | 150 |
| aadA5 | F:CTTCAGTTCGGTGAGTGGC  R:AGGTCGTCGTCCTCGCTAC | 398 |  | Int1 | F: CCTCCCGCACGATGATC  R: TCCACGCATCGTCAGGC | 280 |
| sul3 | F:ATGAGCAAGATTTTTGGAATCGTAA  R:CTAACCCTAGGGCTTTGGATATTT | 792 |  | catl | F: CAATGAAAGACGGTGAGC  R: ACAAACGGCATGATGAAC | 362 |
| TEM-1 | F:TTTTCGTGTCGCCCTTAT  R:GCTCGTCGTTTGGTATGG | 512 |  | cmlA6 | F: GTACGACAGCGAGCACAA  R: CCACCTCCCAGTAGAACG | 124 |
| TEM-206 | F:TGAATGAAGCCATACCAA  R:AGATAACTACGATACGGGAG | 279 |  | floR | F: CTGAACACGACGCCCGCTAT  R: GGACCGCTCCGCAAACAA | 751 |
| blaCTX | F:CGCTTTGCGATGTGCAG  R:ACCGCGATATCGTTGGT | 550 |  | mPrF | F: GGATGCAGGTTTGGTCTG  R: TCGTCAATACGGTTCTCACT | 282 |
| CTXM-55 | F:GCTGGGTAAAGCATTGGG  R:TAGGTTGAGGCTGGGTGA | 224 |  | qnrS | F: ACGACATTCGTCAACTGCAA  R: TAAATTGGCACCCTGTAGGC | 417 |
| AAC(3)-Iia | F:TCACGAACTCGGTCACGC  R:CCAAGCATCGGCATCTCA | 177 |  | blaSHV | F: GGGTTATTCTTATTTGTCGC  R: TTAGCGTTGCCAGTGCTC | 567 |
| aadA2 | F: CATCCCGTGGCGTTATCC  R: CTGGGCAGGTAGGCGTTT | 370 |  | qnrB | F: GATCGTGAAAGCCAGAAAGG  R: ACGATGCCTGGTAGTTGTCC | 469 |
| aadA | F: ATCTGGCTATCTTGCTGACA  R: TTGGTGATCTCGCCTTTC | 388 |  | qnrC | F:ATTTCTCACAGGCAAACT  R: CTGGAATAACAATCACCC | 666 |
| tetA | F: GCTACATCCTGCTTGCCTTC  R: CATAGATCGCCGTGAAGAGG | 210 |  | qnrD | F:TTTTCGCTAACTAACTCGC  R:GAAAGGATAAACAGGCAAAT | 984 |
| 16S rRNA | F: AGAGTTTGATCCTGGCTCAG  R: GGTTACCTTGTTACGACTT | 1369 |  |  |  |  |

Table S3 Drug resistance genes carried by strain 24

| Position | ARO-name | Gene-list |  | Position | ARO-name | Gene-list |
| --- | --- | --- | --- | --- | --- | --- |
| Chromosomal genomes | emrA | chr_1150 |  | Chromosomal genomes | mdtA | chr_1828 |
| Chromosomal genomes | emrB | chr_1149 |  | Chromosomal genomes | mdtB | chr_1827 |
| Chromosomal genomes | emrK | chr_1516 |  | Chromosomal genomes | mdtC | chr_1826 |
| Chromosomal genomes | acrB | chr_3577 |  | Chromosomal genomes | mdtE | chr_214 |
| Chromosomal genomes | TolC | chr_686 |  | Chromosomal genomes | mdtF | chr_213 |
| Chromosomal genomes | emrY | chr_1517 |  | Chromosomal genomes | sdiA | chr_1978 |
| Chromosomal genomes | marA | chr_2373 |  | Chromosomal genomes | baeR | chr_1823 |
| Chromosomal genomes | acrD | chr_1434 |  | Chromosomal genomes | baeS | chr_1824 |
| Chromosomal genomes | AcrE | chr_475 |  | Chromosomal genomes | cpxA | chr_4589 |
| Chromosomal genomes | AcrF | chr_474 |  | Chromosomal genomes | evgA | chr_1515 |
| Chromosomal genomes | gadX | chr_211 |  | Chromosomal genomes | evgS | chr_1514 |
| Chromosomal genomes | emrR | chr_1151 |  | Chromosomal genomes | mdtH | chr_2900 |
| Chromosomal genomes | CRP | chr_402 |  | Chromosomal genomes | MdtK | chr_1917 |
| Chromosomal genomes | macB | chr_3121 |  | Chromosomal genomes | Escherichia coli mdfA | chr_3163 |
| Chromosomal genomes | AcrS | chr_476 |  | Chromosomal genomes | mdtG | chr_2912 |
| Chromosomal genomes | H-NS | chr_1167 |  | Chromosomal genomes | mdtA | chr_1828 |
| Chromosomal genomes | H-NS | chr_2675 |  | Chromosomal genomes | mdtB | chr_1827 |
| Chromosomal genomes | rosA | chr_3560 |  | Chromosomal genomes | eptA | chr_4389 |
| Chromosomal genomes | rosB | chr_3561 |  | Chromosomal genomes | ugd | chr_777 |
| Chromosomal genomes | smeR | chr_4079 |  | Chromosomal genomes | ugd | chr_1871 |
| Chromosomal genomes | dfrA3 | chr_4028 |  | Chromosomal genomes | PmrF | chr_1614 |
| Chromosomal genomes | Escherichia coli rpoB mutants conferring resistance to rifampicin | chr_4516 |  | Chromosomal genomes | basR | chr_696 |
| Chromosomal genomes | Escherichia coli gyrB conferring resistance to aminocoumarin | chr_4 |  | Chromosomal genomes | Klebsiella mutant PhoP conferring antibiotic resistance to colistin | chr_2834 |
| Chromosomal genomes | Escherichia coli parE conferring resistance to fluoroquinolones | chr_691 |  | Chromosomal genomes | Staphylococcus aureus fusA with mutation conferring resistance to fusidic acid | chr_420 |
| Chromosomal genomes | Escherichia coli EF-Tu mutants conferring resistance to Pulvomycin | chr_421 |  | Chromosomal genomes | Escherichia coli nfsA mutations conferring resistance to nitrofurantoin | chr_3154 |
| Chromosomal genomes | Escherichia coli EF-Tu mutants conferring resistance to Enacyloxin IIa | chr_4523 |  | Chromosomal genomes | Escherichia coli murA with mutation conferring resistance to fosfomycin | chr_545 |
| Chromosomal genomes | Escherichia coli AcrAB-TolC with MarR mutations conferring resistance to ciprofloxacin and tetracycline | chr_2374 |  | Chromosomal genomes | Escherichia coli AcrAB-TolC with AcrR mutation conferring resistance to ciprofloxacin, tetracycline, and ceftazidime | chr_3575 |
| Chromosomal genomes | Escherichia coli soxR with mutation conferring antibiotic resistance | chr_4435 |  | Chromosomal genomes | gadW | chr_212 |
| Chromosomal genomes | Salmonella serovars soxS with mutation conferring antibiotic resistance | chr_3786 |  | Chromosomal genomes | kdpE | chr_3318 |
| Chromosomal genomes | Klebsiella aerogenes Omp36 | chr_2445 |  | Chromosomal genomes | leuO | chr_4001 |
| Chromosomal genomes | Klebsiella aerogenes Omp36 | chr_2959 |  | Chromosomal genomes | Escherichia coli GlpT with mutation conferring resistance to fosfomycin | chr_1630 |
| Chromosomal genomes | Escherichia coli folP with mutation conferring resistance to sulfonamides | chr_557 |  | Chromosomal genomes | Escherichia coli UhpT with mutation conferring resistance to fosfomycin | chr_40 |
| Chromosomal genomes | Escherichia coli ompF with mutation conferring resistance to beta-lactam antibiotics | chr_3816 |  | Chromosomal genomes | Escherichia coli uhpA with mutation conferring resistance to fosfomycin | chr_37 |
| Chromosomal genomes | Escherichia coli ompF with mutation conferring resistance to beta-lactam antibiotics | chr_3074 |  | Chromosomal genomes | Escherichia coli PtsI with mutation conferring resistance to fosfomycin | chr_1478 |
| Chromosomal genomes | Mycobacterium tuberculosis rpsL mutations conferring resistance to Streptomycin | chr_418 |  | Chromosomal genomes | Escherichia coli cyaA with mutation conferring resistance to fosfomycin | chr_4698 |
| Chromosomal genomes | Escherichia coli soxS with mutation conferring antibiotic resistance | chr_4081 |  | Chromosomal genomes | rpsJ | chr_425 |
| Chromosomal genomes | Escherichia coli soxS with mutation conferring antibiotic resistance | chr_4436 |  | Chromosomal genomes | Shigella flexneri parC conferring resistance to fluoroquinolones | chr_702 |
| Chromosomal genomes | mdtN | chr_4415 |  | Chromosomal genomes | msbA | chr_3089 |
| Chromosomal genomes | mdtO | chr_4416 |  | Chromosomal genomes | YojI | chr_1660 |
| Chromosomal genomes | mdtP | chr_4417 |  | Chromosomal genomes | hmrM | chr_2222 |
| Chromosomal genomes | LpxA | chr_3901 |  | Chromosomal genomes | Escherichia coli acrA | chr_3576 |
| Chromosomal genomes | LpxC | chr_3982 |  | Chromosomal genomes | Escherichia coli fabI mutations conferring resistance to isoniazid and triclosan | chr_2622 |
| Chromosomal genomes | Escherichia coli fabG mutations conferring resistance to triclosan | chr_2871 |  | Chromosomal genomes | ArnT | chr_1611 |
| Chromosomal genomes | Pseudomonas aeruginosa CpxR | chr_4588 |  | Chromosomal genomes | LptD | chr_4022 |
| Chromosomal genomes | porin OmpC | chr_1656 |  | Chromosomal genomes | ParR | chr_2280 |
| Chromosomal genomes | Klebsiella pneumoniae OmpK37 | chr_2575 |  | Chromosomal genomes | rsmA | chr_1142 |
| Chromosomal genomes | Klebsiella pneumoniae OmpK37 | chr_1932 |  | Chromosomal genomes | Thermus thermophilus uL3 mutations conferring resistance to pleuromutilin antibiotics | chr_426 |
| Chromosomal genomes | Escherichia coli LamB | chr_4462 |  | Chromosomal genomes | Mycobacterium tuberculosis katG mutations conferring resistance to prothionamide | chr_4555 |
| Chromosomal genomes | Escherichia coli mipA | chr_2108 |  | Chromosomal genomes | EC-8 | chr_4351 |
| Chromosomal genomes | Serratia marcescens Omp1 | chr_3484 |  | Chromosomal genomes | Klebsiella pneumoniae mutant PhoQ conferring resistance to colistin | chr_2835 |
| Chromosomal genomes | Mycobacterium tuberculosis thyA with mutation conferring resistance to para-aminosalicylic acid | chr_1009 |  | Chromosomal genomes | Klebsiella pneumoniae PBP3 mutants conferring resistance to ceftazidime-avibactam | chr_3994 |
| Chromosomal genomes | Escherichia coli gyrA with mutation conferring resistance to triclosan | chr_1641 |  | Chromosomal genomes | Bacillus subtilis rpsE mutations conferring resistance to spectinomycin | chr_443 |
| Chromosomal genomes | Klebsiella pneumoniae KpnE | chr_2288 |  | Chromosomal genomes | MdtQ | chr_1729 |
| Chromosomal genomes | Klebsiella pneumoniae KpnF | chr_2289 |  | Chromosomal genomes | ArnT | chr_172 |
| Chromosomal genomes | Clostridioides difficile rpoC with mutation conferring resistance to vancomycin | chr_4515 |  | Chromosomal genomes | LptD | chr_1611 |
| Plasmid 1 genomes | tet(A) | plasmid1_92 |  | Plasmid 1 genomes | APH(6)-Id | plasmid1_89 |
| Plasmid 1 genomes | tet(A) | plasmid1_193 |  | Plasmid 1 genomes | tetR | plasmid1_192 |
| Plasmid 1 genomes | sul2 | plasmid1_87 |  | Plasmid 1 genomes | tetR | plasmid1_91 |
| Plasmid 1 genomes | rmtB | plasmid1_97 |  | Plasmid 1 genomes | oqxA | plasmid1_82 |
| Plasmid 1 genomes | TEM-1 | plasmid1_195 |  | Plasmid 1 genomes | oqxB | plasmid1_83 |
| Plasmid 1 genomes | TEM-1 | plasmid1_96 |  | Plasmid 1 genomes | TEM-244 | plasmid1_99 |
| Plasmid 1 genomes | CTX-M-55 | plasmid1_101 |  | Plasmid 2 genomes | CTX-M-55 | plasmid2_42 |

Table S4 Drug resistance genes carried by strain 27

| Position | ARO-name | Gene-list |  | Position | ARO-name | Gene-list |
| --- | --- | --- | --- | --- | --- | --- |
| Chromosomal genomes | emrA | chr_1140 |  | Chromosomal genomes | H-NS | chr_1155 |
| Chromosomal genomes | emrB | chr_1139 |  | Chromosomal genomes | H-NS | chr_2534 |
| Chromosomal genomes | tet(A) | chr_816 |  | Chromosomal genomes | mdtA | chr_1689 |
| Chromosomal genomes | tet(B) | chr_1877 |  | Chromosomal genomes | mdtB | chr_1688 |
| Chromosomal genomes | emrK | chr_1416 |  | Chromosomal genomes | mdtC | chr_1687 |
| Chromosomal genomes | acrB | chr_3336 |  | Chromosomal genomes | mdtE | chr_263 |
| Chromosomal genomes | TolC | chr_726 |  | Chromosomal genomes | mdtF | chr_262 |
| Chromosomal genomes | emrY | chr_1417 |  | Chromosomal genomes | sdiA | chr_1868 |
| Chromosomal genomes | marA | chr_2258 |  | Chromosomal genomes | baeR | chr_1684 |
| Chromosomal genomes | sul2 | chr_2621 |  | Chromosomal genomes | baeS | chr_1685 |
| Chromosomal genomes | sul2 | chr_811 |  | Chromosomal genomes | cpxA | chr_4312 |
| Chromosomal genomes | acrD | chr_1328 |  | Chromosomal genomes | evgA | chr_1415 |
| Chromosomal genomes | AcrE | chr_502 |  | Chromosomal genomes | evgS | chr_1414 |
| Chromosomal genomes | AcrF | chr_501 |  | Chromosomal genomes | mdtM | chr_3861 |
| Chromosomal genomes | gadX | chr_260 |  | Chromosomal genomes | mdtH | chr_2703 |
| Chromosomal genomes | emrR | chr_1141 |  | Chromosomal genomes | MdtK | chr_1796 |
| Chromosomal genomes | CRP | chr_414 |  | Chromosomal genomes | Escherichia coli mdfA | chr_2978 |
| Chromosomal genomes | macB | chr_2922 |  | Chromosomal genomes | mdtG | chr_2715 |
| Chromosomal genomes | APH(3'')-Ib | chr_2620 |  | Chromosomal genomes | Escherichia coli folP with mutation conferring resistance to sulfonamides | chr_589 |
| Chromosomal genomes | APH(3'')-Ib | chr_812 |  | Chromosomal genomes | Escherichia coli ompF with mutation conferring resistance to beta-lactam antibiotics | chr_1819 |
| Chromosomal genomes | APH(3')-Ia | chr_2617 |  | Chromosomal genomes | Escherichia coli ompF with mutation conferring resistance to beta-lactam antibiotics | chr_3567 |
| Chromosomal genomes | APH(6)-Id | chr_813 |  | Chromosomal genomes | Escherichia coli ompF with mutation conferring resistance to beta-lactam antibiotics | chr_2830 |
| Chromosomal genomes | APH(6)-Id | chr_2619 |  | Chromosomal genomes | Mycobacterium tuberculosis rpsL mutations conferring resistance to Streptomycin | chr_430 |
| Chromosomal genomes | floR | chr_818 |  | Chromosomal genomes | tetR | chr_815 |
| Chromosomal genomes | arnA | chr_1511 |  | Chromosomal genomes | tetR | chr_1878 |
| Chromosomal genomes | bacA | chr_706 |  | Chromosomal genomes | Escherichia coli soxS with mutation conferring antibiotic resistance | chr_3812 |
| Chromosomal genomes | rosA | chr_3319 |  | Chromosomal genomes | Escherichia coli soxS with mutation conferring antibiotic resistance | chr_4157 |
| Chromosomal genomes | rosB | chr_3320 |  | Chromosomal genomes | Escherichia coli soxS with mutation conferring antibiotic resistance | chr_1875 |
| Chromosomal genomes | smeR | chr_3810 |  | Chromosomal genomes | mdtN | chr_4136 |
| Chromosomal genomes | dfrA3 | chr_3757 |  | Chromosomal genomes | mdtO | chr_4137 |
| Chromosomal genomes | Escherichia coli rpoB mutants conferring resistance to rifampicin | chr_4243 |  | Chromosomal genomes | mdtP | chr_4138 |
| Chromosomal genomes | Escherichia coli gyrB conferring resistance to aminocoumarin | chr_4 |  | Chromosomal genomes | LpxA | chr_3629 |
| Chromosomal genomes | Escherichia coli parE conferring resistance to fluoroquinolones | chr_731 |  | Chromosomal genomes | LpxC | chr_3711 |
| Chromosomal genomes | Escherichia coli EF-Tu mutants conferring resistance to Pulvomycin | chr_433 |  | Chromosomal genomes | eptA | chr_4102 |
| Chromosomal genomes | Escherichia coli EF-Tu mutants conferring resistance to Enacyloxin IIa | chr_4250 |  | Chromosomal genomes | ugd | chr_1722 |
| Chromosomal genomes | Escherichia coli AcrAB-TolC with MarR mutations conferring resistance to ciprofloxacin and tetracycline | chr_2259 |  | Chromosomal genomes | PmrF | chr_1512 |
| Chromosomal genomes | Escherichia coli soxR with mutation conferring antibiotic resistance | chr_4156 |  | Chromosomal genomes | basR | chr_736 |
| Plasmid 1 genomes | Salmonella serovars soxS with mutation conferring antibiotic resistance | chr_3490 |  | Chromosomal genomes | Klebsiella mutant PhoP conferring antibiotic resistance to colistin | chr_2639 |
| Chromosomal genomes | Staphylococcus aureus fusA with mutation conferring resistance to fusidic acid | chr_432 |  | Chromosomal genomes | Escherichia coli LamB | chr_4193 |
| Chromosomal genomes | Escherichia coli nfsA mutations conferring resistance to nitrofurantoin | chr_2950 |  | Chromosomal genomes | Escherichia coli mipA | chr_2007 |
| Chromosomal genomes | Escherichia coli murA with mutation conferring resistance to fosfomycin | chr_577 |  | Chromosomal genomes | Mycobacterium tuberculosis thyA with mutation conferring resistance to para-aminosalicylic acid | chr_999 |
| Chromosomal genomes | Escherichia coli AcrAB-TolC with AcrR mutation conferring resistance to ciprofloxacin, tetracycline, and ceftazidime | chr_3334 |  | Chromosomal genomes | Escherichia coli ampC beta-lactamase | chr_4066 |
| Chromosomal genomes | gadW | chr_261 |  | Chromosomal genomes | Escherichia coli gyrA with mutation conferring resistance to triclosan | chr_1539 |
| Chromosomal genomes | kdpE | chr_3125 |  | Chromosomal genomes | Klebsiella pneumoniae KpnE | chr_2190 |
| Chromosomal genomes | leuO | chr_3730 |  | Chromosomal genomes | Klebsiella pneumoniae KpnF | chr_2191 |
| Chromosomal genomes | Escherichia coli GlpT with mutation conferring resistance to fosfomycin | chr_1530 |  | Chromosomal genomes | Clostridioides difficile rpoC with mutation conferring resistance to vancomycin | chr_4242 |
| Chromosomal genomes | Escherichia coli UhpT with mutation conferring resistance to fosfomycin | chr_39 |  | Chromosomal genomes | Mycobacterium tuberculosis rpsA mutations conferring resistance to pyrazinamide | chr_2848 |
| Chromosomal genomes | Escherichia coli uhpA with mutation conferring resistance to fosfomycin | chr_36 |  | Chromosomal genomes | Neisseria gonorrhoeae rpld | chr_453 |
| Chromosomal genomes | Escherichia coli PtsI with mutation conferring resistance to fosfomycin | chr_1372 |  | Chromosomal genomes | YajC | chr_3391 |
| Chromosomal genomes | Escherichia coli cyaA with mutation conferring resistance to fosfomycin | chr_4419 |  | Chromosomal genomes | mlaF | chr_571 |
| Chromosomal genomes | rpsJ | chr_451 |  | Chromosomal genomes | mlaD | chr_573 |
| Chromosomal genomes | Shigella flexneri parC conferring resistance to fluoroquinolones | chr_742 |  | Chromosomal genomes | OmpA | chr_2803 |
| Chromosomal genomes | msbA | chr_2845 |  | Chromosomal genomes | eptB | chr_227 |
| Chromosomal genomes | YojI | chr_1558 |  | Chromosomal genomes | ArnT | chr_1509 |
| Chromosomal genomes | hmrM | chr_2124 |  | Chromosomal genomes | LptD | chr_3751 |
| Chromosomal genomes | Escherichia coli emrE | chr_1850 |  | Chromosomal genomes | ParR | chr_2182 |
| Chromosomal genomes | Escherichia coli acrA | chr_3335 |  | Chromosomal genomes | rsmA | chr_1134 |
| Chromosomal genomes | Escherichia coli fabI mutations conferring resistance to isoniazid and triclosan | chr_2477 |  | Chromosomal genomes | Thermus thermophilus uL3 mutations conferring resistance to pleuromutilin antibiotics | chr_452 |
| Chromosomal genomes | Escherichia coli fabG mutations conferring resistance to triclosan | chr_2676 |  | Chromosomal genomes | Mycobacterium tuberculosis katG mutations conferring resistance to prothionamide | chr_4281 |
| Chromosomal genomes | Pseudomonas aeruginosa CpxR | chr_4311 |  | Chromosomal genomes | Klebsiella pneumoniae mutant PhoQ conferring resistance to colistin | chr_2640 |
| Chromosomal genomes | porin OmpC | chr_1554 |  | Chromosomal genomes | Klebsiella pneumoniae PBP3 mutants conferring resistance to ceftazidime-avibactam | chr_3723 |
| Chromosomal genomes | Klebsiella pneumoniae OmpK37 | chr_2417 |  | Chromosomal genomes | Bacillus subtilis rpsE mutations conferring resistance to spectinomycin | chr_469 |
| Chromosomal genomes | MdtQ | chr_1628 |  | Plasmid 1 genomes | sul2 | plasmid1_139 |
| Plasmid 1 genomes | CTX-M-14 | plasmid1_126 |  | Plasmid 3 genomes | sul1 | plasmid3_48 |
| Plasmid 1 genomes | AAC(3)-IVa | plasmid1_132 |  | Plasmid 3 genomes | sul1 | plasmid3_53 |
| Plasmid 1 genomes | APH(4)-Ia | plasmid1_133 |  | Plasmid 3 genomes | aadA16 | plasmid3_46 |
| Plasmid 1 genomes | dfrA17 | plasmid1_154 |  | Plasmid 3 genomes | QnrB6 | plasmid3_51 |
| Plasmid 1 genomes | FosA3 | plasmid1_128 |  | Plasmid 3 genomes | arr-3 | plasmid3_44 |
| Plasmid 1 genomes | oqxA | plasmid1_158 |  | Plasmid 3 genomes | tetR | plasmid3_9 |
| Plasmid 1 genomes | oqxB | plasmid1_157 |  | Plasmid 3 genomes | dfrA27 | plasmid3_45 |
| Plasmid 3 genomes | tet(A) | plasmid3_10 |  | Plasmid 3 genomes | AAC(6')-Ib-cr6 | plasmid3_43 |

Table S5 Drug resistance genes carried by strain 36

| Position | ARO-name | Gene-list |  | Position | ARO-name | Gene-list |
| --- | --- | --- | --- | --- | --- | --- |
| Chromosomal genomes | emrA | chr_1067 |  | Chromosomal genomes | mdtC | chr_1674 |
| Chromosomal genomes | emrB | chr_1066 |  | Chromosomal genomes | mdtE | chr_266 |
| Chromosomal genomes | emrK | chr_1402 |  | Chromosomal genomes | mdtF | chr_265 |
| Chromosomal genomes | acrB | chr_3360 |  | Chromosomal genomes | sdiA | chr_1851 |
| Chromosomal genomes | TolC | chr_732 |  | Chromosomal genomes | baeR | chr_1671 |
| Chromosomal genomes | emrY | chr_1403 |  | Chromosomal genomes | baeS | chr_1672 |
| Chromosomal genomes | marA | chr_2238 |  | Chromosomal genomes | cpxA | chr_4297 |
| Chromosomal genomes | sul2 | chr_2630 |  | Chromosomal genomes | evgA | chr_1401 |
| Chromosomal genomes | acrD | chr_1314 |  | Chromosomal genomes | evgS | chr_1400 |
| Chromosomal genomes | AcrE | chr_508 |  | Chromosomal genomes | mdtM | chr_3829 |
| Chromosomal genomes | AcrF | chr_507 |  | Chromosomal genomes | mdtH | chr_2714 |
| Chromosomal genomes | gadX | chr_263 |  | Chromosomal genomes | MdtK | chr_1774 |
| Chromosomal genomes | emrR | chr_1068 |  | Chromosomal genomes | Escherichia coli mdfA | chr_2946 |
| Chromosomal genomes | CRP | chr_419 |  | Chromosomal genomes | mdtG | chr_2726 |
| Chromosomal genomes | macB | chr_2891 |  | Chromosomal genomes | APH(3'')-Ib | chr_2629 |
| Chromosomal genomes | H-NS | chr_1082 |  | Chromosomal genomes | APH(3')-Ia | chr_2626 |
| Chromosomal genomes | H-NS | chr_2543 |  | Chromosomal genomes | APH(6)-Id | chr_2628 |
| Chromosomal genomes | mdtA | chr_1676 |  | Chromosomal genomes | arnA | chr_1497 |
| Chromosomal genomes | mdtB | chr_1675 |  | Chromosomal genomes | bacA | chr_712 |
| Chromosomal genomes | rosA | chr_3343 |  | Chromosomal genomes | mdtN | chr_4103 |
| Chromosomal genomes | rosB | chr_3344 |  | Chromosomal genomes | mdtO | chr_4104 |
| Chromosomal genomes | smeR | chr_3778 |  | Chromosomal genomes | mdtP | chr_4105 |
| Chromosomal genomes | dfrA3 | chr_3725 |  | Chromosomal genomes | LpxA | chr_3597 |
| Chromosomal genomes | Escherichia coli rpoB mutants conferring resistance to rifampicin | chr_4227 |  | Chromosomal genomes | LpxC | chr_3679 |
| Chromosomal genomes | Escherichia coli gyrB conferring resistance to aminocoumarin | chr_4 |  | Chromosomal genomes | eptA | chr_4069 |
| Chromosomal genomes | Escherichia coli parE conferring resistance to fluoroquinolones | chr_737 |  | Chromosomal genomes | ugd | chr_1714 |
| Chromosomal genomes | Escherichia coli EF-Tu mutants conferring resistance to Pulvomycin | chr_438 |  | Chromosomal genomes | PmrF | chr_1498 |
| Chromosomal genomes | Escherichia coli EF-Tu mutants conferring resistance to Enacyloxin IIa | chr_4234 |  | Chromosomal genomes | basR | chr_742 |
| Chromosomal genomes | Escherichia coli AcrAB-TolC with MarR mutations conferring resistance to ciprofloxacin and tetracycline | chr_2239 |  | Chromosomal genomes | Klebsiella mutant PhoP conferring antibiotic resistance to colistin | chr_2650 |
| Chromosomal genomes | Escherichia coli soxR with mutation conferring antibiotic resistance | chr_4123 |  | Chromosomal genomes | Staphylococcus aureus fusA with mutation conferring resistance to fusidic acid | chr_437 |
| Chromosomal genomes | Salmonella serovars soxS with mutation conferring antibiotic resistance | chr_3515 |  | Chromosomal genomes | Escherichia coli nfsA mutations conferring resistance to nitrofurantoin | chr_2919 |
| Chromosomal genomes | Escherichia coli folP with mutation conferring resistance to sulfonamides | chr_595 |  | Chromosomal genomes | Escherichia coli murA with mutation conferring resistance to fosfomycin | chr_583 |
| Chromosomal genomes | Escherichia coli ompF with mutation conferring resistance to beta-lactam antibiotics | chr_3535 |  | Chromosomal genomes | Escherichia coli AcrAB-TolC with AcrR mutation conferring resistance to ciprofloxacin, tetracycline, and ceftazidime | chr_3358 |
| Chromosomal genomes | Escherichia coli ompF with mutation conferring resistance to beta-lactam antibiotics | chr_2842 |  | Chromosomal genomes | gadW | chr_264 |
| Chromosomal genomes | Mycobacterium tuberculosis rpsL mutations conferring resistance to Streptomycin | chr_435 |  | Chromosomal genomes | kdpE | chr_3094 |
| Chromosomal genomes | Escherichia coli soxS with mutation conferring antibiotic resistance | chr_3780 |  | Chromosomal genomes | leuO | chr_3698 |
| Chromosomal genomes | Escherichia coli soxS with mutation conferring antibiotic resistance | chr_4124 |  | Chromosomal genomes | Escherichia coli GlpT with mutation conferring resistance to fosfomycin | chr_1516 |
| Chromosomal genomes | Escherichia coli soxS with mutation conferring antibiotic resistance | chr_1858 |  | Chromosomal genomes | Escherichia coli UhpT with mutation conferring resistance to fosfomycin | chr_41 |
| Chromosomal genomes | Escherichia coli uhpA with mutation conferring resistance to fosfomycin | chr_38 |  | Chromosomal genomes | Escherichia coli gyrA with mutation conferring resistance to triclosan | chr_1525 |
| Chromosomal genomes | Escherichia coli PtsI with mutation conferring resistance to fosfomycin | chr_1358 |  | Chromosomal genomes | Klebsiella pneumoniae KpnE | chr_2172 |
| Chromosomal genomes | Escherichia coli cyaA with mutation conferring resistance to fosfomycin | chr_4404 |  | Chromosomal genomes | Klebsiella pneumoniae KpnF | chr_2173 |
| Chromosomal genomes | rpsJ | chr_456 |  | Chromosomal genomes | Clostridioides difficile rpoC with mutation conferring resistance to vancomycin | chr_4226 |
| Chromosomal genomes | Shigella flexneri parC conferring resistance to fluoroquinolones | chr_748 |  | Chromosomal genomes | Mycobacterium tuberculosis rpsA mutations conferring resistance to pyrazinamide | chr_2860 |
| Chromosomal genomes | msbA | chr_2857 |  | Chromosomal genomes | Neisseria gonorrhoeae rpld | chr_458 |
| Chromosomal genomes | YojI | chr_1544 |  | Chromosomal genomes | YajC | chr_3416 |
| Chromosomal genomes | hmrM | chr_2106 |  | Chromosomal genomes | mlaF | chr_577 |
| Chromosomal genomes | Escherichia coli acrA | chr_3359 |  | Chromosomal genomes | mlaD | chr_579 |
| Chromosomal genomes | Escherichia coli fabI mutations conferring resistance to isoniazid and triclosan | chr_2486 |  | Chromosomal genomes | OmpA | chr_2815 |
| Chromosomal genomes | Escherichia coli fabG mutations conferring resistance to triclosan | chr_2687 |  | Chromosomal genomes | eptB | chr_230 |
| Chromosomal genomes | Pseudomonas aeruginosa CpxR | chr_4296 |  | Chromosomal genomes | ArnT | chr_1495 |
| Chromosomal genomes | porin OmpC | chr_1540 |  | Chromosomal genomes | LptD | chr_3719 |
| Chromosomal genomes | Klebsiella pneumoniae OmpK37 | chr_2426 |  | Chromosomal genomes | ParR | chr_2164 |
| Chromosomal genomes | Klebsiella pneumoniae OmpK37 | chr_1806 |  | Chromosomal genomes | rsmA | chr_1061 |
| Chromosomal genomes | Escherichia coli LamB | chr_4175 |  | Chromosomal genomes | Thermus thermophilus uL3 mutations conferring resistance to pleuromutilin antibiotics | chr_457 |
| Chromosomal genomes | Escherichia coli mipA | chr_1988 |  | Chromosomal genomes | Mycobacterium tuberculosis katG mutations conferring resistance to prothionamide | chr_4266 |
| Chromosomal genomes | Mycobacterium tuberculosis thyA with mutation conferring resistance to para-aminosalicylic acid | chr_928 |  | Chromosomal genomes | Klebsiella pneumoniae mutant PhoQ conferring resistance to colistin | chr_2651 |
| Chromosomal genomes | Escherichia coli ampC beta-lactamase | chr_4032 |  | Chromosomal genomes | Klebsiella pneumoniae PBP3 mutants conferring resistance to ceftazidime-avibactam | chr_3691 |
| Chromosomal genomes | Bacillus subtilis rpsE mutations conferring resistance to spectinomycin | chr_474 |  | Chromosomal genomes | MdtQ | chr_1615 |
| Plasmid 1 genomes | mphA | plasmid1_81 |  | Plasmid 2 genomes | mphA | plasmid2_25 |
| Plasmid 1 genomes | sul1 | plasmid1_73 |  | Plasmid 2 genomes | sul1 | plasmid2_17 |
| Plasmid 1 genomes | macB | plasmid1_131 |  | Plasmid 2 genomes | CTX-M-55 | plasmid2_31 |
| Plasmid 1 genomes | TEM-1 | plasmid1_60 |  | Plasmid 2 genomes | aadA16 | plasmid2_15 |
| Plasmid 1 genomes | aadA5 | plasmid1_71 |  | Plasmid 2 genomes | arr-3 | plasmid2_13 |
| Plasmid 1 genomes | dfrA17 | plasmid1_70 |  | Plasmid 2 genomes | FosA3 | plasmid2_89 |
| Plasmid 1 genomes | Mrx | plasmid1_80 |  | Plasmid 2 genomes | Mrx | plasmid2_24 |
| Plasmid 1 genomes | oqxA | plasmid1_88 |  | Plasmid 2 genomes | dfrA27 | plasmid2_14 |
| Plasmid 1 genomes | oqxB | plasmid1_87 |  | Plasmid 2 genomes | AAC(3)-IId | plasmid2_8 |
| Plasmid 1 genomes | AAC(3)-IIe | plasmid1_83 |  | Plasmid 2 genomes | AAC(6')-Ib-cr6 | plasmid2_12 |
| Plasmid 1 genomes | qacEdelta1 | plasmid1_72 |  | Plasmid 2 genomes | TEM-244 | plasmid2_29 |
| Plasmid 5 genomes | tet(A) | plasmid5_1 |  | Plasmid 5 genomes | tetR | plasmid5_6 |

Table S6 Drug resistance genes carried by strain 15

| Position | ARO-name | Gene-list |  | Position | ARO-name | Gene-list |
| --- | --- | --- | --- | --- | --- | --- |
| Chromosomal genomes | emrA | GE001096 |  | Chromosomal genomes | mdtE | GE000241 |
| Chromosomal genomes | emrB | GE001095 |  | Chromosomal genomes | mdtF | GE000240 |
| Chromosomal genomes | emrK | GE001368 |  | Chromosomal genomes | baeR | GE001764 |
| Chromosomal genomes | acrB | GE003657 |  | Chromosomal genomes | baeS | GE001765 |
| Chromosomal genomes | TolC | GE000703 |  | Chromosomal genomes | cpxA | GE004747 |
| Chromosomal genomes | emrY | GE001369 |  | Chromosomal genomes | evgA | GE001367 |
| Chromosomal genomes | marA | GE002391 |  | Chromosomal genomes | evgS | GE001366 |
| Chromosomal genomes | acrD | GE001283 |  | Chromosomal genomes | mdtM | GE004282 |
| Chromosomal genomes | AcrE | GE000484 |  | Chromosomal genomes | mdtH | GE002880 |
| Chromosomal genomes | AcrF | GE000483 |  | Chromosomal genomes | Escherichia coli mdfA | GE003188 |
| Chromosomal genomes | gadX | GE000237 |  | Chromosomal genomes | mdtG | GE002892 |
| Chromosomal genomes | emrR | GE001097 |  | Chromosomal genomes | vgaC | GE005059 |
| Chromosomal genomes | CRP | GE000407 |  | Chromosomal genomes | bacA | GE000682 |
| Chromosomal genomes | AcrS | GE000485 |  | Chromosomal genomes | Escherichia coli EF-Tu mutants conferring resistance to Pulvomycin | GE000426GE004636 |
| Chromosomal genomes | H-NS | GE002658 |  | Chromosomal genomes | Escherichia coli marR mutant conferring antibiotic resistance | GE002392 |
| Chromosomal genomes | mdtA | GE001769 |  | Chromosomal genomes | Escherichia coli soxR with mutation conferring antibiotic resistance | GE004546 |
| Chromosomal genomes | mdtB | GE001768 |  | Chromosomal genomes | Escherichia coli soxS with mutation conferring antibiotic resistance | GE004547 |
| Chromosomal genomes | mdtC | GE001767 |  | Chromosomal genomes | mdtN | GE004526 |
| Chromosomal genomes | emrA | GE001096 |  | Chromosomal genomes | mdtO | GE004527 |
| Chromosomal genomes | emrB | GE001095 |  | Chromosomal genomes | mdtE | GE000241 |
| Chromosomal genomes | emrK | GE001368 |  | Chromosomal genomes | mdtF | GE000240 |
| Chromosomal genomes | acrB | GE003657 |  | Chromosomal genomes | baeR | GE001764 |
| Chromosomal genomes | TolC | GE000703 |  | Chromosomal genomes | baeS | GE001765 |
| Chromosomal genomes | mdtP | GE004528 |  | Chromosomal genomes | LpxC | chr_3679 |
| Chromosomal genomes | eptA | GE004493 |  | Chromosomal genomes | eptA | chr_4069 |
| Chromosomal genomes | ugd | GE001811 |  | Chromosomal genomes | ugd | chr_1714 |
| Chromosomal genomes | PmrF | GE001601 |  | Chromosomal genomes | PmrF | chr_1498 |
| Chromosomal genomes | Escherichia coli nfsA mutations conferring resistance to nitrofurantoin | GE003139 |  | Chromosomal genomes | basR | chr_742 |
| Chromosomal genomes | Escherichia coli acrR with mutation conferring multidrug antibiotic resistance | GE003655 |  | Chromosomal genomes | Klebsiella mutant PhoP conferring antibiotic resistance to colistin | chr_2650 |
| Chromosomal genomes | kdpE | GE003403 |  | Chromosomal genomes | Staphylococcus aureus fusA with mutation conferring resistance to fusidic acid | chr_437 |
| Chromosomal genomes | Escherichia coli GlpT with mutation conferring resistance to fosfomycin | GE001612 |  | Chromosomal genomes | Escherichia coli nfsA mutations conferring resistance to nitrofurantoin | chr_2919 |
| Chromosomal genomes | msbA | GE003077 |  | Chromosomal genomes | Escherichia coli murA with mutation conferring resistance to fosfomycin | chr_583 |
| Chromosomal genomes | YojI | GE001634 |  | Chromosomal genomes | Escherichia coli AcrAB-TolC with AcrR mutation conferring resistance to ciprofloxacin, tetracycline, and ceftazidime | chr_3358 |
| Chromosomal genomes | Escherichia coli acrA | GE003656 |  | Chromosomal genomes | Haemophilus influenzae PBP3 conferring resistance to beta-lactam antibiotics | GE003982 |
| Chromosomal genomes | Escherichia coli ampC beta-lactamase | GE004454 |  | Plasmid 2 genomes | vgaC | GE005059 |

Table S7 Mobile elements carrying drug resistance genes in strain 24

| Position | Composite transposon | Insertion sequence | Family | Strand | Resistance gene |
| --- | --- | --- | --- | --- | --- |
| Chromosomal genomes | No | Yes | IS3 | reverse | marA |
| Plasmid 1 genomes | No | Yes | IS6 | reverse | TEM-1、TEM-244、tet(A)、APH(3’)-Ib、APH(6)-Id |

Table S8 Mobile elements carrying drug resistance genes in strain 27

| Position | Composite transposon | Insertion sequence | Family | Strand | Resistance gene |
| --- | --- | --- | --- | --- | --- |
| Chromosomal genomes | No | Yes | IS66 | reverse | marA |
| Plasmid 1 genomes | No | Yes | IS6 | forward | APH(4’)-Ia、sul2、AAC(3)-Ⅳa、dfrA17 |

Table S9 Mobile elements carrying drug resistance genes in strain 36

| Position | Composite transposon | Insertion sequence | Family | Strand | Resistance gene |
| --- | --- | --- | --- | --- | --- |
| Chromosomal genomes | No | Yes | IS66 | reverse | marA |
| Plasmid 1 genomes | No | Yes | IS1 | forward | aadA5、dfrA17、sul1、TEM-1、AAC(3)-IIe、mphA |
| Plasmid 2 genomes | No | Yes | IS6 | reverse | mphA、oqxB、macB、oqxA、mphA |
